# Unusual C΄/D΄ motifs enable box C/D snoRNPs to modify multiple sites in the same rRNA target region

**DOI:** 10.1093/nar/gkw842

**Published:** 2016-09-19

**Authors:** Robert Willem van Nues, Nicholas James Watkins

**Affiliations:** Institute for Cell and Molecular Biology, Newcastle University, Newcastle upon Tyne, NE2 4HH, UK

## Abstract

Eukaryotic box C/D small nucleolar (sno)RNPs catalyse the site-specific 2΄-*O*-methylation of ribosomal RNA. The RNA component (snoRNA) contains guide regions that base-pair with the target site to select the single nucleotide to be modified. The terminal C/D and internal C΄/D΄ motifs in the snoRNA, adjacent to the guide region, function as binding sites for the snoRNP proteins including the enzymatic subunit fibrillarin/Nop1. Four yeast snoRNAs are unusual in that they are predicted to methylate two nucleotides in a single target region. In each case, the internal C΄/D΄ motifs from these snoRNAs differ from the consensus. Our data indicate that the C΄/D΄ motifs in snR13, snR48 and U18 form two alternative structures that lead to differences in the position of the proteins bound to this motif. We propose that each snoRNA forms two different snoRNPs, subtly different in how the proteins are bound to the C΄/D΄ motif, leading to 2΄-*O*-methylation of different nucleotides in the target region. For snR48 and U18, the unusual C΄/D΄ alone is enough for the modification of two nucleotides. However, for the snR13 snoRNA the unusual C΄/D΄ motif and extra base-pairing, which stimulates rRNA 2΄-*O*-methylation, are both critical for multiple modifications in the target region.

## INTRODUCTION

Ribosomal RNA (rRNA) is highly modified with the two most frequent modifications being 2΄-*O*-methyl groups and pseudouridine (Ψ) (1). 2΄-*O*-methylation can stabilise single base-pairs or hydrogen bonds and can strengthen or alter RNA folds while Ψ stabilises specific RNA structures (2,3). 2΄-O-methylation is found in almost all types of RNA in the cell, including tRNAs, mRNAs, small nuclear (sn)RNAs and micro (mi)RNAs (4). The majority of the 2΄-*O*-methyl groups are clustered in functionally important regions of the ribosome such as the peptidyl transferase domain, the decoding centre and the intersubunit bridge (1). The rRNA modification clusters are essential for cell growth (5–10) while loss of some individual modifications increases cellular sensitivity to stress and ribosome-specific antibiotics (11). The specificity of modification is important as 2΄-*O*-methylation of the wrong site can affect ribosome formation and/or function (12–14). Furthermore, with the recent observations that some sites in the rRNA are not 2΄-*O*-methylated to a 100% (15,16) and the interest in ‘specialised’ ribosomes (17), there is renewed interest in the mechanism controlling the complexity of rRNA modification.

**Figure 1.**
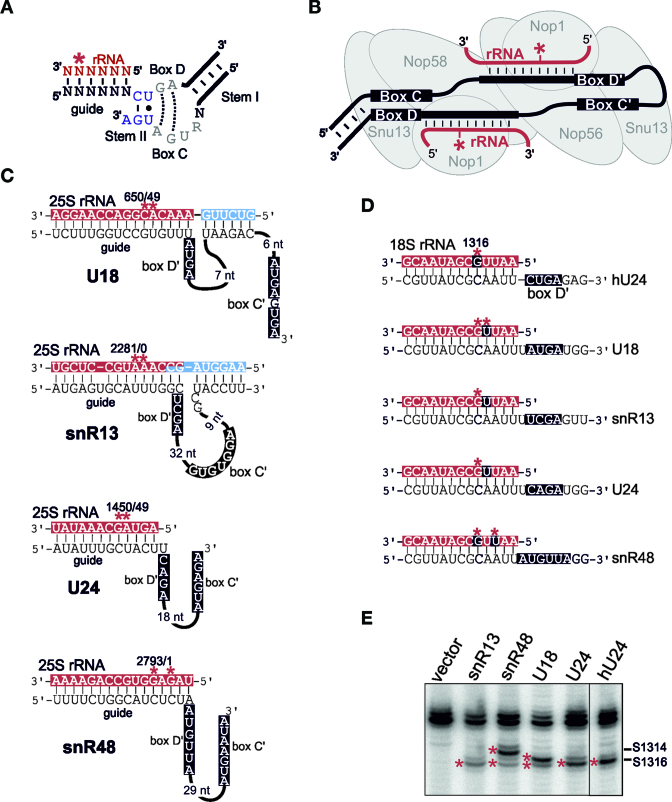
The 2΄-*O*-methylation activity of snR13, snR48, U18 and U24 C΄/D΄ motifs. (**A**) Schematic representation of the box C/D motif with the adjacent guide sequence base-paired to the rRNA target (shown in red with the target nucleotide indicated by an asterisk). The sequence of the C and D boxes are shown. The positions of stem I and II in the motif are indicated. Note that the C/D motif is rotated 180° relative to its orientation in (B) so that it is in the same orientation as the C΄/D΄ motif. (**B**) A schematic model of the box C/D snoRNP complex. The snoRNA is shown in black, with the C, D, C΄ and D΄ boxes indicated. The rRNA is shown in red, with the base-pairing interactions indicated. The asterisk represents the site modified. The box C/D snoRNP proteins are represented as grey filled ellipses. (**C**) rRNA (upper) and snoRNA (lower) sequences, with both conventional guide-rRNA interactions (rRNA shaded red) and novel extra (or accessory) base-pairing (rRNA shaded blue) interactions, for the *S. cerevisiae* box C/D snoRNAs that direct multi-site modification. Where sequences are shaded both red and blue, this indicates an overlap between the conventional and extra-base-pairing. The C΄ and D΄ sequences are shown in white with a black background. The guide sequences in the snoRNA are indicated. Note for snR48 there is no obvious D΄ box and the whole conserved area is marked (see later). The modified nucleotides are indicated by an asterisk together with the rRNA nucleotide number. (**D**) The guide sequence and D΄ motif of the artificial snoRNA constructs used to test the function of the various C΄/D΄ motifs is shown (the full sequence used is shown in Supplementary Figure S1B and C). The D΄ boxes are presented in white with a black background. The target 18S sequence (white text on red background) is shown with the expected (black background) and actual (asterisk) methylation sites indicated. (**E**) Constructs expressing artificial snoRNAs containing the C΄/D΄ motifs from U18, U24, snR13, snR48 and human (h)U24 (as indicated above each lane) were transformed into yeast cells. RNA was extracted from the cells and analysed by primer extension to detect rRNA methylation. The position of the stop corresponding to methylation of the target nucleotides, S1314 and S1316 in the 18S rRNA, are indicated on the left. Bands corresponding to 2΄-*O*-methylation are indicated by an asterisk. Northern blotting was used to control for snoRNA expression (Supplementary Figure S2A).

In eukaryotes and archaea, 2΄-*O-*methylation is catalysed by box C/D snoRNPs and the related box C/D sRNPs, respectively (18). The RNA components of these complexes (snoRNAs and sRNAs) contain a conserved C/D motif at the termini of the RNA and an internal C΄/D΄ motif (Figure 1A). Both motifs share the same consensus, although the C/D motif is part of a stem-internal loop-stem structure, also known as a K-turn (Figure 1A), while the C΄/D΄ motif often forms a stem-loop structure, lacking stem I, which is known as a K-loop (18). The snoRNA/sRNA functions as a guide for modification by base-pairing to the target site with the nucleotide in the snoRNA–rRNA duplex five base-pairs upstream of box D or D΄ being 2΄-*O*-methylated (Figure 1A; (18)). The eukaryotic snoRNPs contain four common core proteins, Snu13 (15.5K in humans), Nop56, Nop58 and Nop1 (fibrillarin) while the archaeal complexes are associated with L7Ae, Nop5 and fibrillarin. Fibrillarin/Nop1 is the active methyltransferase subunit and L7Ae/Snu13 are the primary RNA-binding proteins that recognise the sheared GA base-pairs at the centre of the C/D and C΄/D΄ motifs (Figure 1B; (18)). Archaeal Nop5 homodimerises and this homodimer forms a structural scaffold that links the proteins bound to the C/D and C΄/D΄ motifs (19–22). In eukaryotes, Nop56 and Nop58, which are homologous to Nop5 are predicted to form a heterodimer that bridges the complex and contacts the C΄/D΄ and C/D motifs, respectively (Figure 1B).

High-resolution structures of assembled archaeal box C/D sRNP have been published (20–22) with some debate as to whether the complex is a dimer or monomer. It is worth noting that the only eukaryotic box C/D snoRNP structure published so far, the U3 snoRNP, is clearly a monomer (23). From the archaeal structures, however, it is clear that L7Ae and Nop5 contact the C/D and C΄/D΄ motifs with the C-terminal domain of Nop5 specifically contacting stem II (22). The N-terminal domain of Nop5 interacts with fibrillarin and this unit, referred to as the catalytic module, is mobile and docks onto the sRNA–rRNA duplex upon substrate binding. Fibrillarin binds nucleotides 1–6 of the guide-substrate duplex and a series of non-specific contacts with L7Ae and Nop5 lead to the correct positioning of the active site of this methyltransferase (22). The flexibility/movement of the catalytic module is likely important for binding and release of the modified rRNA. Based on the sequence homology of the various components and the work performed so far, the eukaryotic box C/D snoRNPs are predicted to have a similar structure to that seen for the archaeal sRNPs (18).

While archaeal sRNAs are compact with highly conserved sequence motifs, the eukaryotic snoRNAs are significantly more varied (18). In archaea the C/D and C΄/D΄ motifs are highly conserved and very similar. In eukaryotes, while the C/D and C΄/D΄ motifs share the same consensus, the C/D motif is considerably more conserved than the C΄/D΄ motif (24). The eukaryotic C΄ box can contain insertions although it is unclear how this would be accommodated with respect to protein-binding (24). Furthermore, several C΄/D΄ motifs that are clearly active in directing 2΄-*O*-methylation, vary considerably from the consensus (24). In eukaryotes, an extra, accessory rRNA base-pairing element, that stimulates snoRNP-catalysed methylation by interacting with the rRNA adjacent to the target region of the snoRNA has been described in more than half of the box C/D snoRNAs (24). In yeast, the U18, U24, snR13 and snR48 snoRNAs have been suggested to modify more than one nucleotide in the target region using a single guide and C΄/D΄ region (Figure 1C). Recent deep-sequencing approaches indicate that, for each snoRNA, both sites are almost completely modified (15,16). In each case, deletion of the snoRNA abolishes both modifications (25,26) and, in the absence of alternative methytransferases, it is likely that the C΄/D΄ motifs of these snoRNAs direct multiple modifications. It is, however, unclear how the specificity of box C/D snoRNP catalysed methylation can be altered to enable the 2΄-*O-*methylation of multiple sites in one target region. We therefore set out to investigate the ability of the U18, U24, snR13 and snR48 snoRNAs to direct multiple modifications in a single target region.

## MATERIALS AND METHODS

### Generation of snoRNA constructs

The artificial snoRNA construct, inserted in the intron of the actin gene and under the control of a GAL1 promoter, in the plasmid pRS416, was reported previously (24). C΄/D΄ regions, and the target site guide, were subsequently assembled from oligonucleotides and cloned into the NheI and MluI sites in the snoRNA coding sequence (Supplementary Figure S1). All experiments using these constructs were performed in *S. cerevisiae* strain W303 (*leu2-3112 trp1-1 can1-100 ura3-1 ade2-1 his3-11,15*).

The snR13 gene was PCR amplified using primers snR13fw (5΄ AATAGGATCCCAACGTGAAGAAGCC 3΄) and snR13rev (5΄ AACAGAATTCGCTTGCTTAGGCCCAAC 3΄) and was cloned into the Acc65I/XhoI sites of pRS416. Plasmids expressing wild-type or mutant snR13 were then transformed into the yeast strain YS630 (*ade2, his3, trp1, ura3, leu2, snR13::TRP)* (27). Mutations in the snR13 coding sequence were generated using PCR-based mutagenesis.

### Analysis of rRNA methylation and snoRNA expression

Methylation activity was determined by reverse transcription under limited nucleotide and enzyme concentrations (28) as described previously (24). A total of 8 μg RNA was annealed to ^32^P-, 5΄-end labelled primer and then incubated with M-MLV reverse transcriptase (40 u, Promega), 2 μl 5xRT buffer, 0.25 μl superasin and 1.25 mmol dNTP's. The reactions were separated on either a 6 or 8% polyacrylamide/7 M urea gel and then visualised using a phosphorimager. Primers used for mapping were Map1316 (5΄-TAGTCCCTCTAAGAAGTGGATAACC-3΄) and Map13 (5΄-CTAGATAGTAGATAGGGACAGTGG-3΄). To determine the expression of snoRNAs, Northern blot analysis was performed with the following probes snR13 (5΄-CCACACCGTTACTGATTTGGCAAAA GC-3΄), snR5 (5΄-TAAGCATGGTAATCCGGAAGATC-3΄), snR87 (5΄-TAGAACATGGCGGTGTTCCAA GTGAT-3΄), artificial snoRNA (5΄-AATTGCGATAACGCTAGCTACATC-3΄) and 5S rRNA (5΄-CTACTCGGTCAGGCTC-3΄) as described previously (24). In some cases, methylene blue staining of 5S rRNA was used to check for equal loading.

## RESULTS

### Non-canonical 2΄-*O*-methylation directed by the C΄/D΄ motifs of snR13, snR48, U18 and U24 snoRNAs

The box C/D or C΄/D΄ motif with an adjacent guide region has been shown to direct the modification of a single nucleotide in the rRNA five base-pairs away from the D/D΄ box (Figure 1A). This is performed by positioning of the active site of Nop1/fibrillarin, which binds the C/D or C΄/D΄ motif, at the nucleotide to be modified (Figure 1B). However, the C΄/D΄ motifs and respective guides of the naturally occurring yeast snR13, snR48, U18 and U24 snoRNAs have been shown to direct the modification of two nucleotides in the same target region of the rRNA (Figure 1C). For U18, U24 and snR13 these nucleotides are adjacent, while for snR48 the nucleotides are separated by one nucleotide. In each case, deletion of the snoRNA results in the loss of both modifications (25,26). The C΄/D΄ motifs of these snoRNAs, each of which deviates from the consensus ((24) C΄ – RUGAUGA; D΄ – CUGA), could result in the modification of multiple sites within the rRNA target region (Figure 1C). It is important to note that while motifs resembling the consensus D΄ motif could be found in snR13, U18 and U24, no such motif could be found for snR48 and the conserved region we have designated the D΄ box (see later) is six nucleotides long. However, it is also possible that other mechanisms, such as a second methytransferase that recognises the primary snoRNP-catalysed modification and methylates after this has occured, could generate the modification profiles seen with the U18, U24, snR13 and snR48 snoRNPs.

To test whether the C΄/D΄ motifs of snR13, snR48, U18 and U24 alone are capable of directing multi-site modification we inserted the C΄/D΄ motifs from these snoRNAs into an artificial snoRNA construct, based on the human U24 snoRNA, that we have previously described (24). In the control construct for these experiments, the C΄/D΄ motif from the human U24 snoRNA (which unlike its yeast counterpart follows the consensus standard) was placed adjacent to a guide sequence that targets nt S1316, an unmodified region of the yeast 18S rRNA (Figure 1D; nt with black background indicates the predicted target site). The C΄/D΄ motifs of snR13, U18 and U24 were cloned into the artificial snoRNA construct to target site S1315 based on the established 5-base-pair rule for 2΄-*O*-methylation (Figure 1D). If the C΄/D΄ motifs are capable of multiple modifications then site S1316 should also be modified. Since snR48 does not contain a recognisable D΄ motif, we cloned the C΄/D΄ motif such that it should, based on snR48 modification of its natural target site, target nucleotides S1316 and S1314. The constructs expressing the artificial snoRNAs were transformed into yeast cells. Northern blotting was used to demonstrate that the snoRNAs were all expressed to the expected levels (Supplementary Figure S2A). The modified nucleotide in the target region of the 18S rRNA was then determined by primer extension under reduced nucleotide concentrations where 2΄-*O*-methylated nucleotides cause the reverse transcriptase to terminate at a higher frequency.

A reverse-transcriptase stop, consistent with 2΄-*O*-methylation, was observed at nucleotide S1316 when the artificial snoRNA containing the human U24 C΄/D΄ motif (hU24) was expressed, but not in the absence of a snoRNA (Figure 1E; methylation bands indicated by asterisk). A weaker stop was frequently observed at nucleotide S1315 one nucleotide above the target site, as we have observed previously (24). It is important to note that we also observed a weak stop one nucleotide above almost all natural methylation sites in the rRNA that we tested. We believe that this additional, weaker band, is due to additional stalling by reverse transcriptase and does not reflect additional methylation. 2΄-*O-*methylation at nucleotide S1316 was also seen when snoRNAs containing the C΄/D΄ motifs of snR13 and U24 were expressed. In contrast, the snoRNA containing the U18 C΄/D΄ motif resulted in methylation of nucleotides S1316 and S1315 while the snR48 C΄/D΄ directed modification at sites S1316 and S1314 (Figure 1E). Our data clearly show that the C΄/D΄ motifs of U18 and snR48 directed the methylation of two distinct nucleotides in the target region. Indeed, the methylation pattern seen for the U18 and snR48 C΄/D΄ motifs replicated the 2΄-*O-*methylation pattern attributed to the full-length snoRNAs (Figure 1C and D). Surprisingly, while multiple-site methylation was not seen with the box C΄/D΄ snoRNA motifs of snR13 and U24, both motifs directed modification of a nucleotide six nucleotides upstream of the D΄ box (Figure 1D). Furthermore, our data suggest that for both U24 and snR13, other elements in the snoRNA, in addition to the C΄/D΄ motif, or possibly an additional trans-acting factor, play a role in the 2΄-*O*-methylation of multiple sites in their target region.

### The sequence of stem II in the U18 C΄/D΄ motif causes the methylation of two adjacent nucleotides in the target region

**Figure 2.**
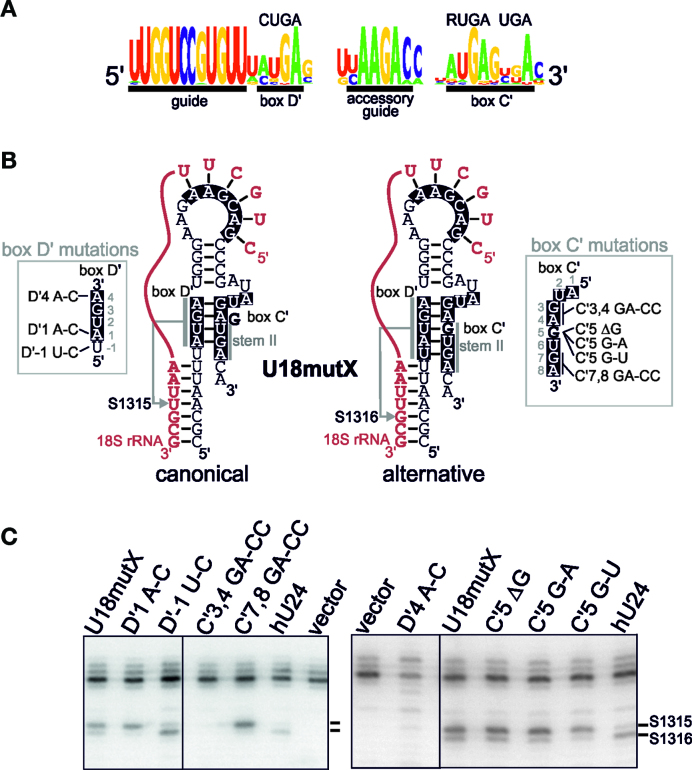
The 5΄ end of box D΄ and the extra nucleotide in box C΄ are both required for multiple site 2΄-*O*-methylation by the U18 snoRNA. (**A**) A WebLogo representation of the evolutionary conservation of the guide, D΄ box, accessory guide and C΄ box sequences (as indicated below) of the U18 snoRNA derived from the alignment of all the available yeast U18 snoRNA sequences (24). The consensus C΄ and D΄ sequences are shown above. The diagram was prepared using the WebLogo software (29). (**B**) Two secondary structure models, one showing canonical base-pairing the other an alternative structure for stem II, of the U18mutX box C΄/D΄ motif and guide sequence, in the context of the artificial snoRNA, base-paired to the 18S rRNA. Note, in the alternative structure a longer, 5 nucleotide box D΄ is used. The U18mutX RNA differs from the wild-type U18 in the sequence of the accessory guide region (Supplementary Figure S1C) that has been altered to base-pair with the 18S rRNA adjacent the region targeted by the artificial snoRNA. The arrows indicate the site modified. The box C΄/D΄ motif and the accessory guide are shown in white on a black background. The positions of the C΄, D΄ boxes and stem II are indicated. The sequence of the box C΄ and D΄ mutants is also shown to the right and left of the structures, respectively. The numbering system used starts at the first position of the canonical box sequence. (**C**) Plasmids expressing artificial snoRNAs containing the wild-type and mutant U18 C΄/D΄ motifs (as indicated above each lane) were transformed into yeast cells. RNA was extracted from the cells and analysed by primer extension to detect rRNA methylation. The position of the stop corresponding to methylation of the target nucleotides, S1315 and S1316 in the 18S rRNA, are indicated. The levels of the various snoRNAs were determined by Northern blotting (Supplementary Figure S2B).

Having shown that the U18 and snR48 C΄/D΄ motifs both direct multi-site 2΄-*O*-methylation, we were next interested in determining why these motifs have this unusual property. Starting first with U18, analysis of the U18 C΄/D΄ motif from all available yeast sequences ((24); Figure 2A) revealed that the D΄ box consensus (AUGA) is close to the canonical D΄ box sequence (CUGA) while the C΄ motif contains a single nucleotide insertion (AUGAGUGA). Analysis of the structure of the U18 C΄/D΄ motif revealed that the main variation is found in the stem II region (Figure 2B). One possible explanation for the 2΄-*O*-methylation of two adjacent nucleotides is that the D΄ region contains two distinct, overlapping D΄ boxes (Figure 2B; left structure AUGA and right structure UAUGA) that would result in two alternative structures for stem II (Figure 2B; canonical and alternative models). In the canonical structure, the extra nucleotide in box C΄ (AUGAGUGA) is not base-paired and bulges out, enabling normal stem II interactions between the two boxes. In the alternative structure model, the extra nucleotide base-pairs with the D΄ box and forms part of stem II. We therefore suggest that the two distinct structures of stem II could result in different positioning of the methyltransferase Nop1 in the complex in each case. The alternative modes of Nop1 binding could therefore place adjacent nucleotides into the active site of Nop1, therefore resulting in the modification of two distinct nucleotides.

To investigate this further, we generated a series of mutants in the U18 C΄/D΄ region in the artificial construct to test the importance of the stem II sequence in multi-site 2΄-O-methylation (Figure 2B). For these experiments we also mutated the extra base-pairing element in the U18 C΄/D΄ such that it base-paired upstream of the 18S rRNA target site to fully replicate the base-pairing interactions seen with the wild-type U18 snoRNA and the 25S rRNA (U18mutX; Figure 2B and Supplementary Figure S1C). It is important to note that we found that the extra base-pairing played no role in multi-site modification or methylation efficiency by the U18C΄/D΄ region (data not shown). The constructs were transformed into yeast cells, the expression of the snoRNAs was monitored by Northern blotting (Supplementary Figure S2B) and the ability to direct 2΄-*O*-methylation was analysed by primer extension.

Mutation of the first GA di-nucleotide in the C΄ box (C΄3,4 GA-CC) abolished methylation at both sites (Figure 2C) while mutation of the last A in the D΄ box (D΄4 A-C) abolished methylation at S1316 and severely reduced methylation at S1315. Interestingly, mutation of the second GA di-nucleotide in box C΄ (C΄7,8 GA-CC) abolished methylation at S1316 but not at S1315. From this we concluded that the GA base-pairs are needed for total activity and that changes to stem II affect the site targeted for methylation. Further mutation of the stem II sequence revealed that deletion of the G insertion (C΄5 ΔG) had little effect on the methylation of S1316 and S1315. However, mutation of this G to a U or A (C΄5 G-U; C΄5 G-A) biased the modification to site S1315 over S1316. Mutation D΄1 A-C, which generates the consensus D΄ box resulted in a reduction in 2΄-*O*-methylation at nucleotide S1316 but no change to methylation at S1315. Conversely, mutation D΄-1 U-C, which places a C at the 3΄ end of the guide region, resulted in methylation of S1316 and a reduction in the signal at S1315 to background levels (Figure 2C; compare D΄-1 U-C to hU24). These data indicate that the sequence of stem II in U18, in particular the 5΄ nt of the D΄ box and the 3΄ nucleotide of the guide, dictate which nucleotides are modified in the target sequence. Importantly, neither of these nucleotides is a C, the consensus nucleotide for the 5΄ end of box D΄, and mutation of either nucleotide to a C dramatically alters the methylation pattern. This therefore implies that the sequence/structure of stem II and, in particular, suboptimal sequences at the 5΄ end of box D΄ can result in the modification of two different nts in the target sequence.

### The unusual D΄ motif in snR48 enables 2΄-*O*-methylation of two nucleotides in the same target region

**Figure 3.**
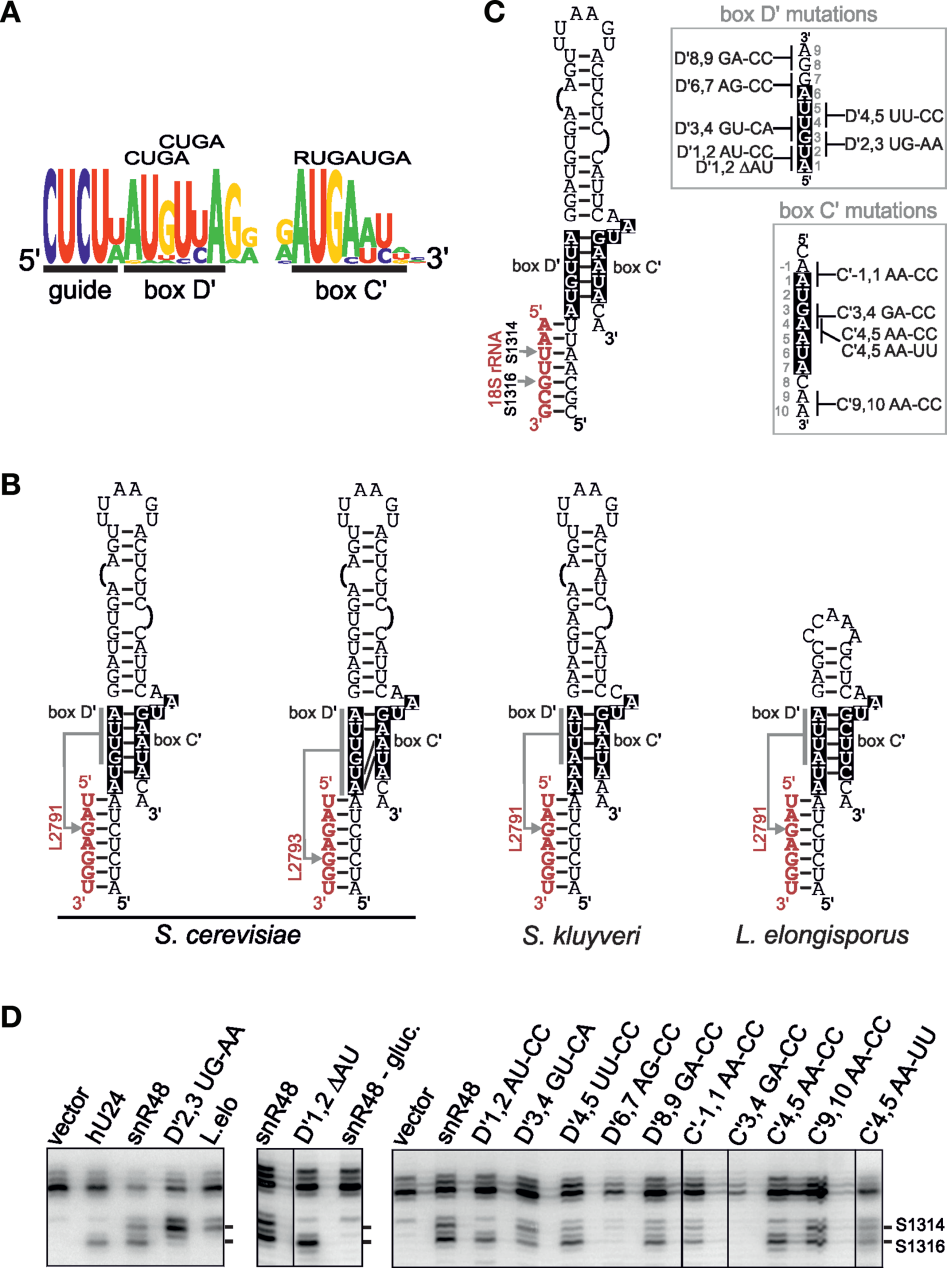
The snR48 C'D' motif is unique and directs 2΄-*O*-methylation at multiple sites in the target region. (**A**) A WebLogo representation of the conservation of the guide, D΄ box and C΄ box sequences of the snR48 snoRNA derived from the alignment of all the available yeast snR48 sequences (24). The consensus sequence for the C΄ and D΄ boxes is shown above the WebLogo image. Note, two potential alternative positions for the D΄ box are shown. The diagram was prepared using the WebLogo software (29). (**B**) Secondary structure of the snR48 box C΄/D΄ motif and guide sequence base-paired to the 25S rRNA from *S. cerevisiae, S. kluyveri* and *L. elongisporus*. Note two alternative structures are shown for the *S. cerevisiae* snR48. The C΄ and D΄ boxes are shown in white on a black background. Arrows indicate the site to be modified. (**C**) Secondary structure of the snR48 box C΄/D΄ motif, in the context of the artificial snoRNA targeting sites 1314 and 1316 in the 18S rRNA. The C΄ and D΄ boxes are shown in white on a black background. Arrows indicate the site to be modified. The mutations to the C΄ and D΄ boxes are shown to the right. (**D**) Constructs expressing artificial snoRNAs containing the wild-type and mutant snR48 C΄/D΄ motifs (as indicated above each lane) were transformed into yeast cells. RNA was extracted from the cells and analysed by primer extension to detect rRNA methylation. The position of the two target nucleotides (S1314 and S1316) is indicated on the right. L.elo is the artificial snoRNA with the C΄/D΄ motif from *L. elongisporus* snR48. snR48 glucose: yeast containing the plasmid encoding the artificial snoRNA with the snR48 C΄/D΄ motif, grown on glucose containing media. The levels of the various snoRNAs were determined by Northern blotting (Supplementary Figure S2C).

Having shown that the unusual C΄/D΄ motif, in particular the D΄ box, in U18 results in the modification of two sites in the target region, we were next interested to determine the elements needed for the unusual activity of the snR48 C΄/D΄ motif. Yeast snR48 guides the 2΄-*O*-methylation of two nucleotides (G2791 and G2793) in the snR48 target region of the 25S rRNA (helix 88 in domain V). Interestingly, by comparing the available yeast RNA sequences we found that the unusual D΄ box region of snR48 is a highly conserved 6 nt region (AUGUUA) that does not contain the CUGA consensus (Figure 3A). Conversely, the C΄ motif, in particular the 5΄ half, fits to the consensus sequence and is as conserved as most yeast C΄ boxes (24). As with U18, we found two possible base-pairing alternatives for the C΄/D΄ motif that may explain the ability of this motif to direct two 2΄-*O*-methylation events in the same target (Figure 3B). This secondary structure is also conserved, with only a couple of exceptions, across a diverse range of fungi. The UG in the D΄ box of *S. cerevisiae* snR48 (from 5΄ positions 2 and 3) is an AA in *S. kluyveri* snR48. This means that only one of the two base-pairing options seen in the *S. cerevisiae* snR48 is possible in the *S. kluyveri* RNA (Figure 3B). In addition, the C΄/D΄ motif in the snR48 snoRNAs from *Lodderomyces elongisporus, Candida parapsilosis, Verticillium dahliae, Phaeosphaeria nodorum, Aspergillus flavus* and *Glomerella graminicola* (Figure 3B and data not shown) also allow for only one of the two base-pairing interactions. To test the methylation activity of the different snR48 C΄/D΄ motifs, we mutated the *S. cerevisiae* sequence in the artificial construct to mimic the sequence of the *S. kluyveri* snR48 D΄ motif (Figure 3C; mutant D΄2,3 UG-AA). In addition, we cloned the C΄/D΄ region from the *L. elongisporus* snR48 snoRNA into the artificial snoRNA construct (Supplementary Figure S1). The ability of these artificial snoRNAs to direct methylation was then analysed as described above.

The use of the *S. kluyveri* D' box resulted in the preferential 2΄-*O*-methyation of site S1314 and all but abolished modification at S1316 (Figure 3D; D΄2,3 UG-AA). This is consistent with the fact that this mutation would interrupt base-pairing with the lower D΄ box and therefore methylation at S1316. Interestingly, reverse transcriptase stops, above background levels, were also seen 1 nt above and below S1314 indicating that the methylation machinery bound to this motif is somewhat promiscuous. The *L. elongisporus* C΄/D΄ motif (Figure 3D; L.elo) also directed modification of S1314 and, to a lesser extent, S1313 but not S1316. This indicates that snR48 C΄/D΄ motifs generally direct the 2΄-*O*-methylation of multiple sites in the target region. However, the pattern of modification in different yeast species can vary from that seen in *S. cerevisiae* depending on the sequence of the C΄/D΄ motif.

We were next interested in analysing the sequence and structural features of the C΄/D΄ motif of the snR48 snoRNA from *S. cerevisiae*. To this end, we generated a series of mutations in the C΄ and D΄ boxes (Figure 3C) and analysed their effect on 2΄-*O*-methylation (Figure 3D). Mutation C΄9,10 AA-CC, which altered nucleotides just downstream of the C΄ box, had no noticeable effect on methylation at either site while the D΄6,7 AG-CC and C΄3,4 GA-CC mutations severely reduced and completely abolished modification at both sites, respectively. The D΄6,7 AG-CC mutation altered the last nucleotide of the predicted D΄ box and suggests that the 3΄ end of the D΄ motif is required for both modification events. A slight reduction in methylation at site S1314 was observed with the D΄5,6 UU-CC and D΄8,9 GA-CC mutations. The D΄1,2 AU-CC and C΄4,5 AA-CC mutations severely reduced 2΄-*O*-methylation at site S1314, while not significantly affecting modification of S1316. Mutations C΄-1,1 AA-CC and C΄4,5 AA-UU reduced methylation at both sites. Interestingly, the C΄4,5 AA-CC and C΄4,5 AA-UU mutations change the same 2 nt but have different outcomes. Deletion of the first 2 nt of box D΄ (Figure 3C; D΄1,2 ΔAU) replaced the AU of the D΄ box with the UU of the guide region and moved the predicted target sites (now S1316 and S1318). This mutant resulted in methylation of S1316 but not S1318 (Figure 3D). Our data indicate that the whole of the D΄ box is essential for the multi-site modification of the target region guided by snR48. However, only the 3΄ A of the D΄ box was absolutely essential for rRNA modification. As with U18, the insertion of C's in the 5΄ end of the D΄ region (mutants D΄1,2 AU-CC) significantly influenced the nucleotide modified. The C΄ box of snR48 appears to be comparable to C΄ boxes found in other yeast snoRNAs. Our data are consistent with snR48 containing two different structures for the C΄/D΄ motif using two overlapping D΄ boxes. However, the interaction between the consensus C΄ box and the D΄ box contains one to two Watson–Crick base-pairs and three non-Watson–Crick base-pairs making it difficult to predict exactly how these boxes and, especially very divergent sequences, interact with each other (see Discussion). This point is exemplified by the fact that the D΄1,2 AU-CC mutation would be predicted to block methylation at S1316, in a similar manner to the D΄1,3 UG-AA mutation, but actually affects 2΄-*O*-methylation at site S1314 and suggests not only that the complete D΄ region is required for both modifications but that a C at the 5΄ end of the D' box determines which nucleotide is modified.

### The last nucleotide of the guide region can influence the site of 2΄-*O*-methylation

**Figure 4.**
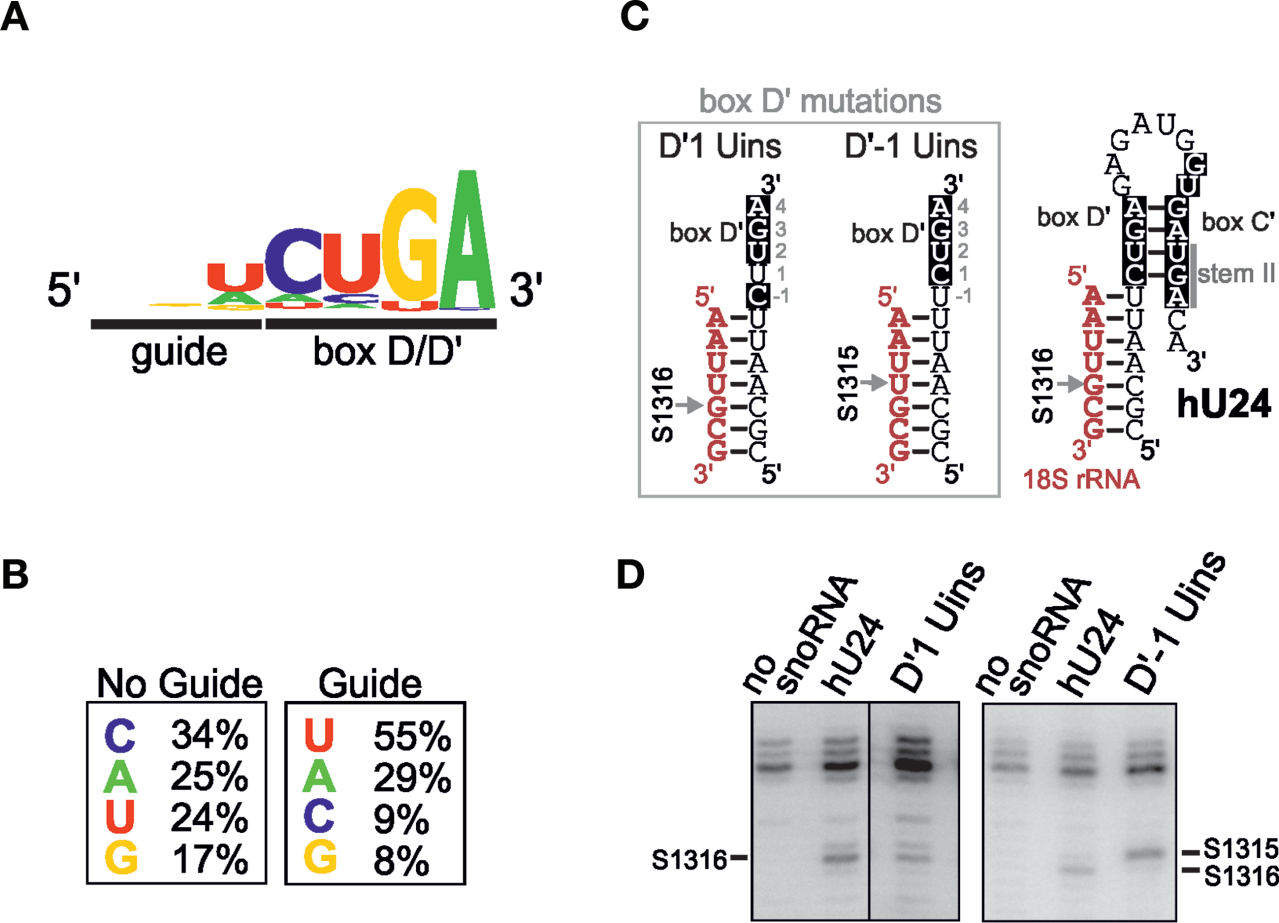
The last nucleotide of the guide region can influence the site to be 2΄-*O*-methylated in the target RNA. (**A**) A schematic representation of the conservation of the active guide and D΄/D box sequences of the *S. cerevisiae* snoRNAs (24). The diagram was prepared using the WebLogo software (29). (**B**) Comparison of the 3΄ nucleotide of the active and inactive (no guide) guide regions of the available yeast box C/D snoRNAs (24). (**C**) Secondary structure of the hU24 box C΄/D΄ motif, in the context of the artificial snoRNA targeting sites 1316 in the 18S rRNA. The C΄ and D΄ boxes are shown in white on a black background. The arrow indicates the site to be modified. The mutations to the D΄ box are shown to the left. (**D**) Constructs expressing artificial snoRNAs containing the wild-type and mutant hU24 C΄/D΄ motifs (as indicated above each lane) were transformed into yeast cells. RNA was extracted from the cells and analysed by primer extension to detect rRNA methylation. The position of the stop corresponding to methylation of the target nucleotides, S1315 and S1316 in the 18S rRNA, are indicated. The levels of the various snoRNAs were determined by Northern blotting (Supplementary Figure S2D).

From the analysis of U18 and snR48, our data indicate that the presence of a C at the 3΄ end of the guide region, particularly where the D΄ box lacks a 5΄ C, can influence the site methylated in the rRNA and extend the D΄ box to five nucleotides. From this we predict that C would not frequently be found at the 3΄ end of the guide region. We therefore compared the sequence of the last three nucleotides of guides, before the D or D΄ box, known to direct 2΄-*O*-methylation in the *S. cerevisiae* snoRNAs. There was no sequence bias in the nucleotides three and two positions before the D/D΄ box (Figure 4A). In contrast, in 60% of the RNAs a U was present in the last position of the guide, A was represented normally, G slightly under-represented and C only present in two cases (Figure 4A). In one case, snR13, the C present at the 3΄ end of the guide is probably part of a longer D΄ box (see later). The data therefore indicate that in *S. cerevisiae*, U is the preferred nucleotide for the last position in the guide region and that C is significantly under-represented. We next compared the sequence of the last nucleotide of the guide region in 2033 active and 556 inactive (i.e. the guide regions that are not conserved and have no obvious target) guides adjacent to the D΄ box for all available yeast snoRNAs that were characterised in our previous bioinformatics analysis (24). While there was a slight bias towards C, and against G, in the inactive guides, this revealed no real significant sequence bias (Figure 4B). However, in the active guides, the sequence was again biased towards U, with C found at this position in only 9% of the snoRNAs. Interestingly, G was also under-represented at this position. These data therefore support our hypothesis that a C at the 3΄ nucleotide of the guide region may interfere with 2΄-*O*-methylation specificity.

We therefore next tested the importance of the last nucleotide of the guide and first nucleotide of the D΄ box in the human U24 C΄/D΄ motif, which modifies a single site when expressed in the context of our artificial snoRNA targeting S1316. Insertion of a U between the C and U of the D΄ box, changing the last nucleotide of the guide to C and the first nucleotide of the D΄ box to U (Figure 4C; D΄1 Uins) should, based on the five nucleotide rule, result in modification of S1315. However, this mutation resulted in the modification of position S1316 (Figure 4D). Importantly, when a U was inserted between the guide and the D΄ box site (Figure 4C; D΄-1 Uins) nucleotide S1315 was modified. This therefore supports our proposal that the last nucleotide of the guide can influence the site to be modified.

### Extra base-pairing and an extended D΄ box are essential for multi-site methylation guided by the snR13 snoRNA

**Figure 5.**
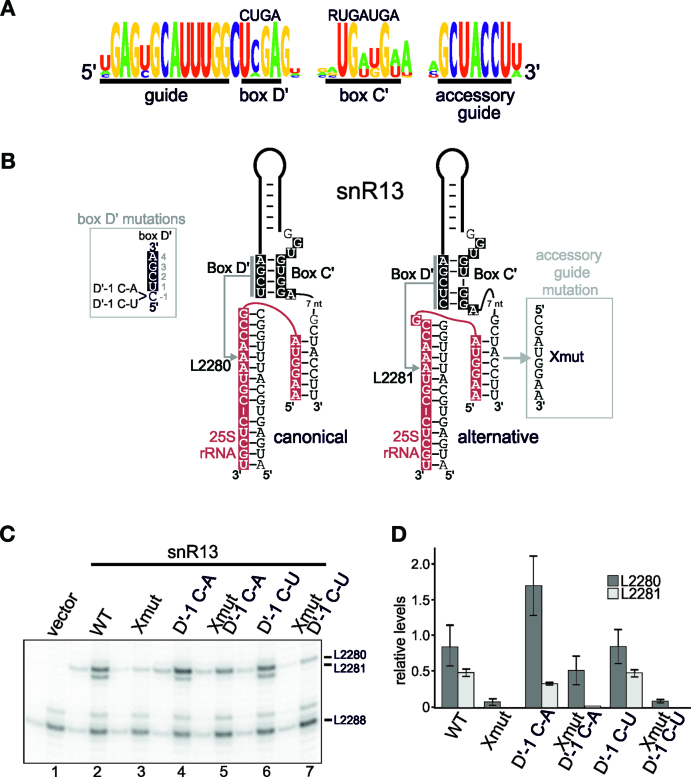
An unusual D΄ box and the extra base-pairing sequence are required for multi-site 2΄-*O*-methylation by the snR13 snoRNA. (**A**) A schematic representation of the conservation of the active guide and D΄/D box sequences of the snR13 snoRNA (24).The consensus for the C΄ and D΄ sequences is shown above the image. The diagram was prepared using the WebLogo software (29). (**B**) Secondary structure models of the snR13 box C΄/D΄ motif, guide region and accessory guide with the natural rRNA target site (shown in red) in the 25S rRNA. The C΄ and D΄ boxes are shown in white on a black background. The arrow indicates the site to be modified. The mutations to the D΄ box are shown to the left and the mutation to the accessory guide is shown on the right. Arrows indicate the site in the rRNA to be modified. Note, in the alternative structure a longer, five nucleotide box D΄ is used. (**C**) Constructs expressing wild-type and mutant snR13 snoRNAs (as indicated above each lane) were transformed into yeast cells. RNA was extracted from the cells and analysed by primer extension to detect rRNA methylation. The position of the stop corresponding to methylation of the target nucleotides, L2280 and L2281 in the 25S rRNA, are indicated. The levels of the various snoRNAs were determined by Northern blotting (Supplementary Figure S2E). (**E**) The data presented in (**D**) was analysed using imagequant software and the relative levels of the bands corresponding to the L2280 and L2281, relative to the signal seen for the modification at L2288, were calculated and plotted. The data were adjusted to the WT signal at L2280. Error bars indicate standard deviation from three separate experiments.

The U24 and snR13 C΄/D΄ motifs both directed methylation six nucleotides from the originally predicted D΄ box (Figure 1E) but neither element, in the context of the artificial snoRNA system, targeted methylation of two adjacent nucleotides. We therefore next investigated whether other elements in the snoRNAs contribute to multi-site modification of the target region. To our surprise, many of the mutations to guide and/or C΄/D΄ regions of yeast U24 frequently affected snoRNA levels to the extent that we could not draw any conclusions from our analysis of this snoRNA (data not shown). We therefore turned our attention to the snR13 snoRNA which directs 2΄-*O*-methylation of nucleotides L2280 and L2281 in the 25S rRNA. snR13 contains an extra base-pairing region or accessory guide (Figure 5A and B; (24)). The snR13 D΄ box is evolutionarily well conserved and again possibly has two D΄ boxes, one four nucleotides (Figure 5B; UCGA) and one five nucleotides in length (CUCGA). Based on the work presented above, the CU present at the 5΄ end of the D΄ box likely contributes to the targeting of the nucleotide six nucleotides into the guide. Given that multi-site modification was not achieved with the artificial snoRNA system, we analysed the role of the extra base-pairing region and the 5΄ end of the D' box in the level and specificity of 2΄-*O*-methylation by the natural snR13 snoRNA. An empty vector or plasmids expressing wild-type snR13 or mutated snR13 (Figure 5B and C), were introduced into a yeast strain where the endogenous snR13 gene had been deleted. RNA was extracted from these strains and 2΄-*O*-methylation was analysed by primer extension.

No 2΄-*O*-methylation of sites L2280 and L2281 was seen in the strain lacking snR13 and methylation was restored at these two sites when the wild-type snR13 was expressed from a plasmid (Figure 5D; compare lanes 1 and 2). Mutation of the nucleotide just before the D΄ box to an A (D΄-1 C-A) resulted in a reproducible increase in modification at site L2280 with a corresponding decrease to modification at site L2281 (Figure 5C (lane 4) and D). Interestingly, this change was not seen when this nucleotide was changed to a U (D΄-1 C-U; lane 6). The C to U change would still allow base-pairing with the second G in the C΄ box (RUGAUGA) while a C to A change would likely abolish this interaction. Mutation of the extra-base-pairing region (Xmut) resulted in a significant reduction of 2΄-*O*-methylation by snR13 at L2280 while modification of nucleotide L2281 was not detectable (lane 3). Interestingly, combining Xmut with the D΄-1 C-A mutation (Xmut-D΄-1 C-A; lane 5) resulted in a slight reduction at modification at site L2280 but a dramatic reduction in 2΄-*O*-methylation at L2281. In contrast, combining Xmut with the D΄-1 C-U mutation gave the same result as the Xmut alone (Xmut-D΄-1 C-U; lane 7). Extra base-pairing can affect the level of methylation (24) but we were surprised to find that this base-pairing interaction can also affect target specificity. Taken together, our data show that the combination of an unusual D΄ box and extra base-pairing lead to the efficient 2΄-*O*-methylation of two sites in the target region by snR13.

## DISCUSSION

Here, we have investigated how a subset of box C/D snoRNAs can modify multiple nucleotides within the rRNA target region using a single guide and C΄/D΄ motif. Our data indicate that this is primarily achieved using unusual C΄/D΄ motifs. The established rule is that the position in the rRNA five nucleotides upstream of the D or D΄ box is methylated (Figure 1A) (25). However, the U18 and snR48 C΄/D΄ motifs, when used in an artificial snoRNA construct, direct the modification of multiple sites in the target region, while the snR13 C΄/D΄ motif directed the modification of the position in the rRNA six nucleotides upstream of the ‘canonical’ D΄ box. The snR48 snoRNA contains a long and unusual D΄ box that lacks a sequence comparable to the consensus and we predict contains both a four and six nucleotide long D΄ motif. In snR13 and U18, the D' motif lacks the C at the 5΄ end (CUGA) which our data indicate is not essential for the level of modification but is important for the correct selection of the target nucleotide. We propose that the snR13 and U18 snoRNAs contain two, overlapping four and five nucleotide long D΄ boxes. The five nucleotide long D΄ boxes include the last (3΄) nucleotide of what is considered the guide region (Figures 2B and 5B). We showed that having a C at the last nucleotide of the guide, which is found naturally in snR13, effectively lengthens the D΄ box by one nucleotide and causes methylation of the target site at a position one nucleotide further away in the target rRNA. Therefore, a C at this position in the guide would be predicted to interfere with target specificity. This is supported by our observation that a C at this position is significantly under-represented in the active guide/D΄ motifs of yeast snoRNAs. The D΄ box has always been considered to function as part of a ‘molecular ruler’ in defining the nucleotide for 2΄-*O*-methylation. Our data, however, indicate that the 5΄ end of the D΄ box, in particular, is the element that determines which nucleotide will be modified. We propose that there are two, overlapping D΄ boxes in snR13, U18 and snR48, which form alternative interactions with the C’ box, and depending on which D΄ box is recognised/used, this determines which nucleotide is modified. One explanation for the ability of these snoRNAs to direct the modification of multiple sites in target region is that each snoRNA can form two structural isoforms, with each capable of modifying a single, distinct, nucleotide. While we believe that the structural isoforms will be stable, especially when the proteins are bound, and two sub-populations of snoRNP exist. However, we cannot rule out the possibility that the snoRNP can alternate between the two conformations.

**Figure 6.**
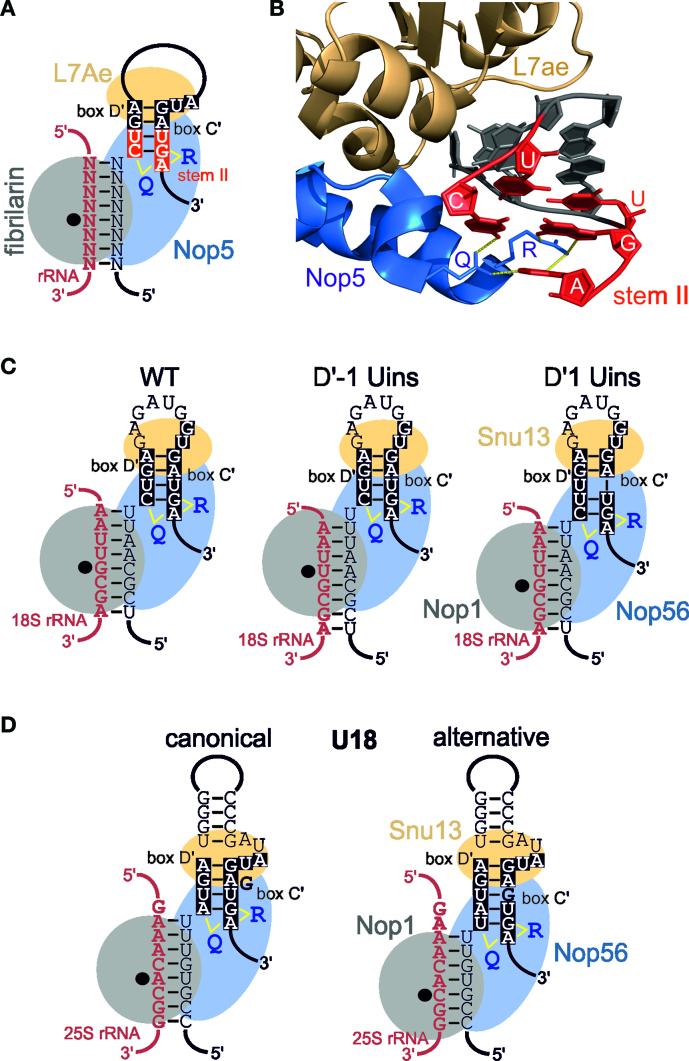
Interaction of Nop56 with stem II of the C΄/D΄ motif dictates the site of 2΄-*O*-methylation. (**A**) Schematic model of the Archaeal C΄/D΄ motif and guide region base-paired to a target rRNA (shown in red). The box C/D snoRNP proteins are represented as grey filled ellipses. The black dot indicates the active site of fibrillarin (Nop1 in yeast) and is positioned over the nucleotide to be modified. The Q and R in Nop5 represent amino acids Q296 and R339 that make contact with stem II of both the C/D and C΄/D΄ motif motifs. Stem II is the C΄/D΄ motif is shown in white on a red background. The upper part of the C΄/D΄ motif, which is bound by L7Ae, is shown in white with a black background. (**B**) Recognition of stem II of the box C/D motif by the Nop domain of Archaeal Nop5 (22). Cartoon views of the relevant regions of Nop5 and L7Ae are shown. Amino acid side chains are only shown for Q296 and R339. The snoRNA is shown in cartoon form and only the C΄/D΄ motif is shown for clarity. Stem II is shown in red and the upper part, bound by L7Ae, is shown in dark grey. The identity of the nucleotides in stem II is indicated. Hydrogen bonds between Q296 and R339 I Nop5 and stem II of the C΄/D΄ motif are shown in yellow. (**C**) Organisation of the snoRNP proteins on the artificial snoRNA containing the hU24 C΄/D΄ motif and the D΄ box insertions presented in Figure 4. The schematic organisation is the same as in (A) except the whole C΄/D΄ motif is shown in white with a black background. Note the slight, one nucleotide shift, in the position of Nop56, and he corresponding change in Nop1 position, in the complex based on the recognition of stem II in the C΄/D΄ motif. (**D**) Organisation of the snoRNP proteins on the U18 snoRNA based on the canonical and alternative secondary structures presented in Figure 2B. The schematic organisation is the same as in (C).

The D΄ box interacts with the C΄ box to form a protein-binding site that functions to position multiple proteins, including the catalytic subunit fibrillarin/Nop1, onto the snoRNA. Based on the structure of the Archaeal box C/D sRNPs (22), both the C/D and C΄/D΄ motifs are bi-partite structures. The GA base-pairs and the first U in the C/C΄ box, form the L7Ae (Snu13 in yeast) binding site (Figure 6A and B). Stem II is bound by Nop5 (Nop58 or Nop56 in yeast). Importantly, the only sequence specific contacts between Nop5 and stem II are between Q296 and R339 in the Nop domain of Nop5 the C in the D/D΄ box and the last GA in the C/C΄ box (Figure 6A and B). Nop5 is homologous to both Nop56 and Nop58 and the Q296 and R339 are evolutionarily highly conserved. We therefore assume that Nop56 would interact with stem II of the eukaryotic C΄/D΄ motif in a similar manner. The catalytic subunit, fibrillarin (Nop1 in yeast) does not contact the C/D or C΄/D΄ motifs and is held in position by its interaction with Nop5. Assuming that this is also the case in eukaryotes, the docking site of Nop56 on the C΄/D΄ motif would therefore dictate where Nop1 is positioned on the substrate RNA (Figure 6A). Based on our data, the C at the 5΄ end of D box appears a major determinant for Nop56 binding and moving this C one nucleotide upstream in the snoRNA causes a similar move in the position of Nop1 on the target rRNA and therefore a one nucleotide shift in the target nucleotide. Figure 6C shows the predicted association of Snu13, Nop56 and Nop1 (fibrillarin) on the artificial snoRNA containing the hU24 C΄/D΄ motif to demonstrate this. Interestingly, only one hydrogen bond is predicted to form between the D΄ box C nucleotide and Nop56, while four hydrogen bonds are expected between Nop56 and the G and A in box C΄. From this we predict that the importance of the C in the D΄ box lies, in part, in positioning the G and A in the C΄ box and therefore creating the Nop56-binding site. Our data indicate that the use of alternative D΄ motifs, which differ in the use of a ‘normal’ and extended D΄ box, accounts for the modification of two nucleotides in the target region by snR13, snR48 and U18. We believe that, for each snoRNA, the cell contains two distinct snoRNPs, which differ in the folding of the C΄/D΄ motif, based on the D΄ box used, which in turn, alters the binding site of Nop56 to the snoRNA. Figure 6D shows the predicted association of Snu13, Nop56 and Nop1 (fibrillarin) with the two alternative structures of the U18 C΄/D΄ motif to illustrate this point. As Nop56 positions Nop1 in the snoRNP, the alternative binding of Nop56 leads to different positioning of Nop1 in the two snoRNPs, relative the rRNA, and therefore results in the modification of different nucleotides in the target region. Furthermore, our data fit nicely with the archaeal structure and support the idea that RNA–protein contacts in the eukaryotic snoRNP will be very similar to those seen in the archaeal box C/D sRNP.

We have also shown that the combination of the unusual C΄/D΄ motif together with the extra base-pairing function together to target the two adjacent nucleotides (L2280 and L2281) in the 25S rRNA by snR13. The extra base-pairing interaction, with it overlapping with the guide region, could also influence the structure of the complex as well as increasing the efficiency of rRNA methylation. However, we have found several instances of overlap between guide and extra-base-pairing interactions in snoRNAs that modify a single site. Extra base-pairing clearly enhances modification at both sites, although the reason why modification at L2281 is more dependent on this than 2΄-*O*-methylation at L2280 is unclear.

Taken together, our data provide clear examples of how one snoRNA can modify multiple nucleotides in the target region by forming two different snoRNP complexes that differ in the positioning of Nop56 and Nop1 (fibrillarin) in the complex. This is different to the normal approach taken by snoRNPs, in which two separate guide regions are used to target different nucleotides. It is clear from our data that the unique D΄ box, in particular with snR13, is evolutionarily conserved and we believe that this scenario is not just found in the *S. cerevisiae* snoRNAs, but will be seen in most yeasts and potentially other eukaryotes. Indeed, evidence suggests that human U24 is also capable of modifying multiple sites within one target region (25). Therefore, with minor changes to the C΄/D΄ motif box C/D snoRNPs can be adapted to increase the complexity of rRNA modification without the need for additional snoRNAs.

## Supplementary Material

Supplementary Data
