# Supplementary material for: Unusual C΄/D΄ motifs enable box C/D snoRNPs to modify multiple sites in the same rRNA target region
Source: Nucleic Acids Res. 2016 Sep 19;45(4):2016–28. doi: 10.1093/nar/gkw842 (PMC5389607; doi:10.1093/nar/gkw842)
Supplement: Supplementary Data [file gkw842_Supp.pdf]

Supplementary Data

Figure S1. snoRNA constructs

A) Schematic representation of the galactose-inducible snoRNA cassette. The positions of the GAL promoter (GALp), ADH terminator sequence (ADHt) and exons 1 and 2 of the actin gene (E1 and E2) are shown. The positions of the NheI and MluI restriction sites, used in the cloning of the various C'/D' fragments, are indicated. **B)** Sequence of the artificial snoRNA cassette showing boxes (indicated above) and restriction sites (indicated below). **C)** Sequence of the different C'/D' motifs from various *S. cerevisiae* snoRNAs that were cloned into the snoRNA cassette. Included is the U18mutX sequence, in which the accessory guide was mutated to base-pair adjacent to the new target region in the 18S rRNA, used in Figure 2. The boxes and accessory guide sequences are indicated. **D)** Sequence of the C'/D' motif from the *L. elongisporous* snR48.

Figure S2. Northern blot analysis of snoRNA levels

RNA was extracted from cells expressing either artificial snoRNA constructs (A), B) C) and D)) or mutant and wild-type snR13 snoRNAs (E), and analysed by Northern hybridisation using probes specific to the snoRNAs of interest (artificial snoRNA, snR13) or loading controls (5S, snR5, snR87). The probe used is indicated on the right and the transformed snoRNA construct indicated above each lane. In panels D and E, methylene blue stained 5S rRNA was used as a loading control.

Figure S1

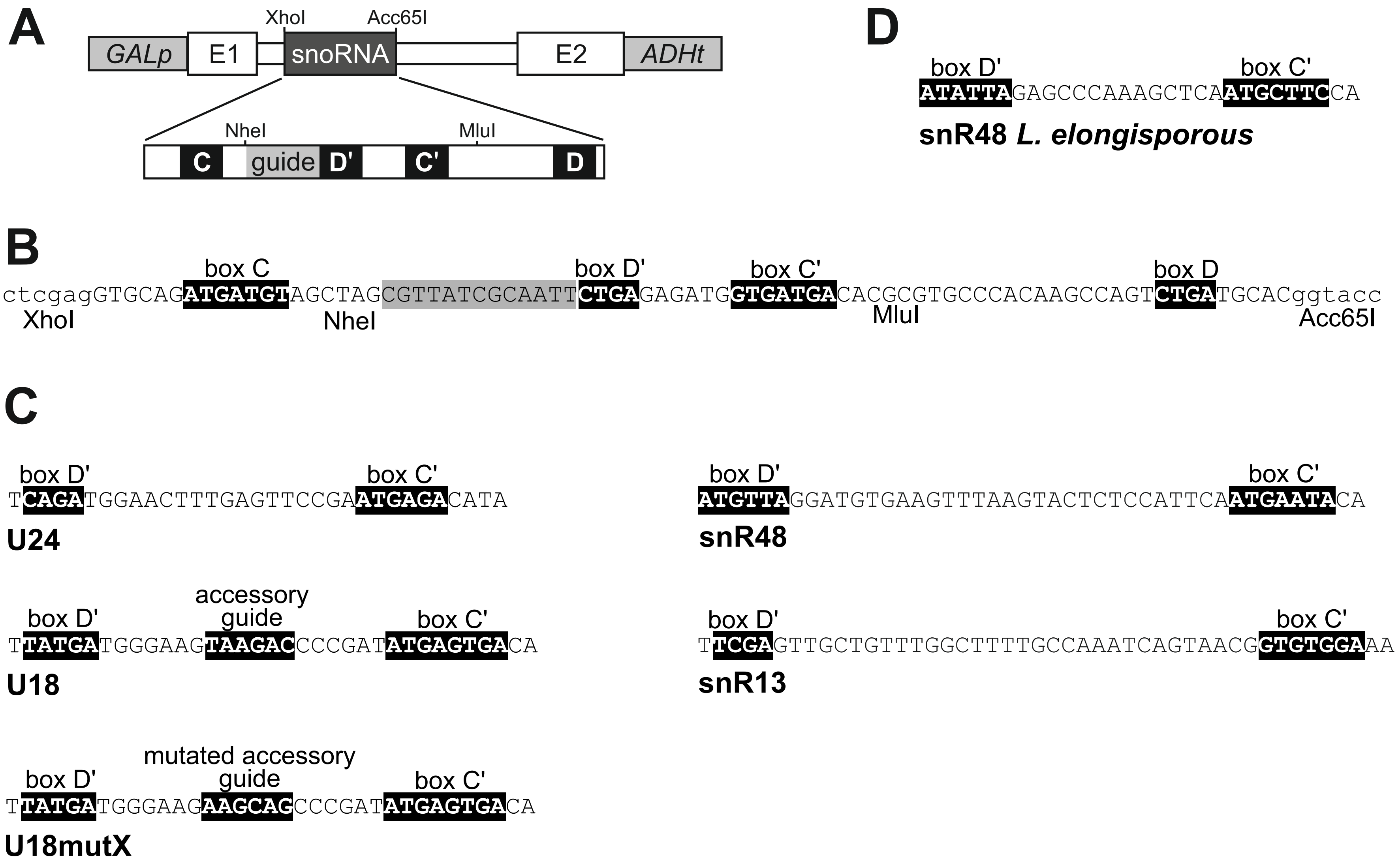

A
XhoI
Acc65I
GALp
E1
snoRNA
E2
ADHt
NheI
MluI
C
guide
D'
C'
D
B
box C
box D'
box C'
box D
ctcgagGTGCAGATGATGTAGCTAGCGTTATCGCAATTCTGAGAGATGGTGATGACACGCGTGCCCACAAGCCAGTCTGATGCACggtacc
XhoI
NheI
MluI
Acc65I
C
box D'
box C'
TCAGATGGAACTTTGAGTTCCGAATGAGACATA
U24
box D'
box C'
ATGTTAGGATGTGAAGTTTAAGTACTCTCCATTCAATGAATACA
snR48
box D'
accessory guide
box C'
TTATGATGGGAAGTAAGACCCCGATATGAGTGACA
U18
box D'
box C'
TTCGAGTTGCTGTTTGGCTTTTGCCAAATCAGTAACGGTGTGGAAA
snR13
box D'
mutated accessory guide
box C'
TTATGATGGGAAGAAGCAGCCCGATATGAGTGACA
U18mutX
D
box D'
box C'
ATATTAGAGCCCAAAGCTCAATGCTTCCA
snR48 L. elongisporous

Figure S2

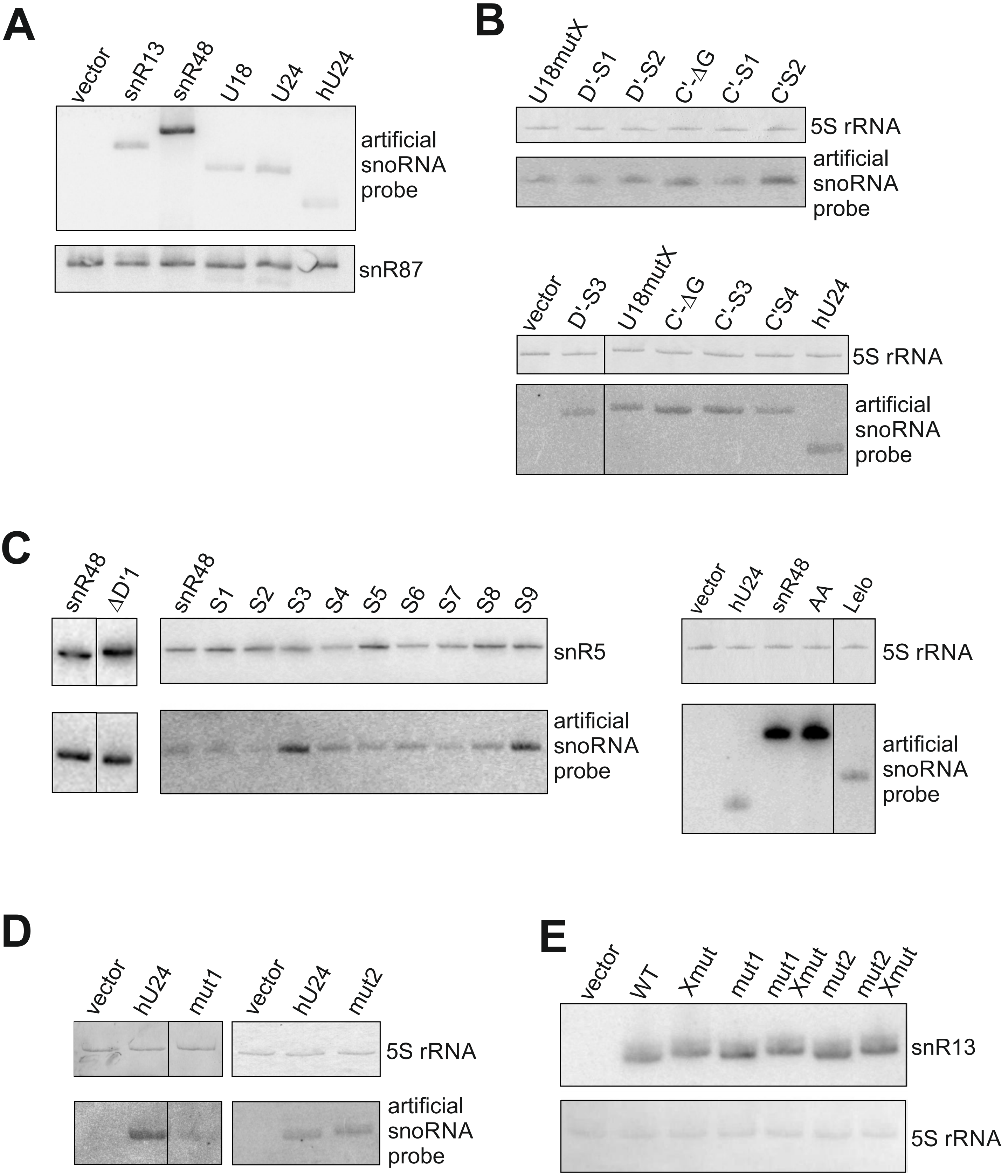
A
vector
snR13
snR48
U18
U24
hU24
artificial snoRNA probe
snR87
B
U18mutX
D'-S1
D'-S2
C'-ΔG
C'-S1
C'S2
5S rRNA
artificial snoRNA probe
vector
D'-S3
U18mutX
C'-ΔG
C'-S3
C'S4
hU24
5S rRNA
artificial snoRNA probe
C
snR48
ΔD'1
snR48
S1
S2
S3
S4
S5
S6
S7
S8
S9
snR5
artificial snoRNA probe
vector
hU24
snR48
AA
Lelo
5S rRNA
artificial snoRNA probe
D
vector
hU24
mut1
vector
hU24
mut2
5S rRNA
artificial snoRNA probe
E
vector
WT
Xmut
mut1
mut1 Xmut
mut2
mut2 Xmut
snR13
5S rRNA
